# Prognostic Value of the RVFWLS/PASP Ratio in Pulmonary Arterial Hypertension

**DOI:** 10.3390/jcdd13040151

**Published:** 2026-03-30

**Authors:** Hongjie Bian, Qinhua Zhao, Fengling Ju, Lan Wang, Yupei Han, Hongling Qiu, Cijun Luo, Pei Gang, Ke Li, Xumeng Ding

**Affiliations:** 1 Nanyang Second General Hospital, Henan Medical University, Nanyang 473000, China; 2 Shanghai Pulmonary Hospital, Tongji University School of Medicine, Shanghai 200433, China

**Keywords:** pulmonary arterial hypertension, right ventricle-pulmonary artery coupling, echocardiography, right ventricular function, prognosis

## Abstract

Background: The right ventricular free wall longitudinal strain to pulmonary arterial systolic pressure (RVFWLS/PASP) ratio is an emerging echocardiographic index for evaluating right ventricular–pulmonary artery (RV-PA) coupling. This study aimed to evaluate its prognostic significance and incremental value in risk stratification for patients with pulmonary arterial hypertension (PAH). Methods: We conducted a retrospective–prospective cohort study of 149 adult PAH patients (87 idiopathic PAH and 62 connective tissue disease-associated PAH). RVFWLS was measured via speckle tracking echocardiography, and PASP was estimated using Doppler. The primary endpoint was event-free survival, defined as the first occurrence of all-cause mortality, lung transplantation, or rehospitalization for right heart failure. Kaplan–Meier and multivariate Cox regression analyses were performed to identify independent predictors. Results: During a median follow-up of 32 months, 78 primary events occurred. Patients in the lower RVFWLS/PASP group (<0.246%/mmHg) exhibited significantly worse exercise capacity, higher NT-proBNP levels, and poorer hemodynamics compared with the higher group (≥0.246%/mmHg) (all *p* < 0.001). The event-free survival rate for the composite endpoint was significantly lower in the group with reduced RVFWLS/PASP compared with that observed in the higher RVFWLS/PASP group (log-rank *p* < 0.05). Multivariate Cox regression analysis demonstrated RVFWLS/PASP ≥ 0.246%/mmHg was independently predictive of reduced risk for the primary endpoint (HR = 0.46, 95%CI 0.23–0.93, *p* < 0.05). Moreover, RVFWLS/PASP facilitated additional risk stratification among patients classified as low risk based on established models (FPHN, COMPERA 2.0, and REVEAL Lite 2). Conclusions: RVFWLS/PASP is a robust, independent determinant of long-term prognosis in patients with PAH. As a noninvasive measure of RV-PA coupling, it provides significant incremental value for clinical risk assessment and treatment monitoring.

## 1. Introduction

Pulmonary arterial hypertension (PAH) is a progressive disease involving both the pulmonary vasculature and the heart. Right ventricular (RV) function is a key determinant of patient symptoms and outcomes [1,2,3,4]. Given that right heart failure in PAH results from increased cardiac afterload, right ventricle–pulmonary artery (RV-PA) coupling provides a more comprehensive framework for understanding RV function [5]. As pulmonary vascular resistance rises, a coupled RV responds by enhancing contractility to match the afterload, thereby preserving RV function. In contrast, a decoupled RV exhibits reduced contractility and fails to adapt to the elevated afterload. Monitoring the level of RV-PA coupling can help predict clinical deterioration in PAH, even during periods of apparent clinical stability.

Echocardiography serves as an essential noninvasive tool for evaluating RV function in patients with PAH [6]. Since conventional parameters for assessing RV function are often influenced by afterload, composite parameters incorporating pulmonary arterial systolic pressure (PASP) may hold greater clinical significance. As a currently widely used parameter of RV-PA coupling, the tricuspid annular plane systolic excursion to PASP ratio (TAPSE/PASP) is recommended for assessing disease severity and risk stratification in all PAH patients [7]. However, this parameter is limited by angle dependence. In contrast, the RV free wall longitudinal strain to PASP ratio (RVFWLS/PASP), derived from speckle tracking echocardiography, directly quantifies RV myocardial deformation in an angle-independent manner, providing a more comprehensive characterization of RV function [2,8].

In this study, we aimed to compare the performance of RVFWLS/PASP with other RV parameters and develop an optimized prognostic model to identify risk profiles in patients with PAH. We hypothesized that RVFWLS/PASP could offer additional prognostic value compared with conventional risk scores.

## 2. Materials and Methods

### 2.1. Study Population and Design

This retrospective–prospective cohort study consecutively enrolled patients with confirmed diagnoses of idiopathic pulmonary arterial hypertension (IPAH) and connective tissue disease-associated pulmonary arterial hypertension (CTD-PAH) from Shanghai Pulmonary Hospital and Nanyang Second People’s Hospital between December 2009 and October 2024. While patient recruitment and baseline clinical data were obtained retrospectively, follow-up for clinical outcomes was conducted prospectively. The study adhered to the ethical principles of the Declaration of Helsinki and was approved by the institutional ethics review board (2024-LY053-01). Due to the ambispective nature of the study, the requirement for written informed consent was waived for patients whose data were collected retrospectively, while verbal or written informed consent was obtained from patients during prospective follow-up, in accordance with the Declaration of Helsinki.

Inclusion criteria required patients to be ≥18 years at diagnosis and meet the 2022 ESC/ERS guideline criteria [7] for PAH confirmed by right heart catheterization (RHC): for IPAH, resting mPAP ≥ 20 mmHg with pulmonary artery wedge pressure (PAWP) ≤ 15 mmHg and pulmonary vascular resistance (PVR) > 2 WU without other known causes; for CTD-PAH, mPAP ≥ 20 mmHg with PAWP ≤ 15 mmHg and PVR > 2 WU with confirmed CTD after excluding other comorbidities. Exclusion criteria included: (1) missing echocardiographic coupling data, (2) unavailable RHC data, (3) incomplete follow-up information. Ultimately, the final study cohort comprised 87 patients with IPAH and 62 with CTD-PAH.

### 2.2. Study Endpoints

The primary endpoint was defined as a composite of clinical worsening. This was operationalized as the first occurrence of any of the following: (1) all-cause mortality, (2) lung transplantation, or (3) rehospitalization due to right heart failure.

Patients were prospectively followed through regular outpatient clinic visits or telephone interviews to record clinical outcomes until the first composite endpoint event, loss to follow-up, or study cut-off date (May 2025), whichever occurred first. Patients who could not be reached were considered lost to follow-up. Follow-up time for survival analysis was calculated from the date of enrollment (baseline echocardiography) to the date of the first qualifying event or censoring, with those lost to follow-up being censored at their last documented clinical contact. For the primary composite endpoint analysis, the total number of events was 78. The composite endpoint was chosen as the primary outcome to enhance statistical power and to comprehensively capture the broad spectrum of disease progression in PAH.

### 2.3. Baseline Data Collection

Baseline data were collected for all enrolled patients by reviewing electronic medical records, including demographic characteristics (age, sex), World Health Organization functional class (WHO-FC), 6 min walking distance (6MWD), and N-terminal pro-brain natriuretic peptide (NT-proBNP). Cardiac output and related pulmonary hemodynamic parameters were measured using a Swan-Ganz catheter equipped with a triple-lumen balloon (Edwards Lifesciences, Irvine, CA, USA) via the thermodilution method [9,10].

### 2.4. Echocardiographic Measurements and Definitions

Baseline echocardiographic measurements were performed within 24–48 h after RHC using commercially available equipment (Vivid 7 and E95; GE Healthcare, Horten, Norway) in standard views, with all data reviewed by at least two echocardiography specialists. Measurements were obtained from the average of three consecutive cardiac cycles according to the American Society of Echocardiography guidelines [11]. RV morphological parameters included RV mid-diameter (RVMD), left ventricular eccentricity index (LV-EId) [12], and right atrial area, while conventional RV functional parameters comprised lateral tricuspid annular systolic velocity (S’), tricuspid annular plane systolic excursion (TAPSE), pulmonary arterial systolic pressure (PASP), left ventricular ejection fraction (LVEF), left ventricular end-diastolic diameter (LVEDD), and tricuspid regurgitation. The tricuspid regurgitation (TR) pressure gradient was calculated from the continuous-wave Doppler TR velocity by the simplified Bernoulli equation. PASP was calculated by the formula PASP = 4 × (peak velocity of TR)^2^ + estimated right atrial pressure. Right atrial pressure was based on inferior vena cava diameter and collapsibility.

**Figure 1:**
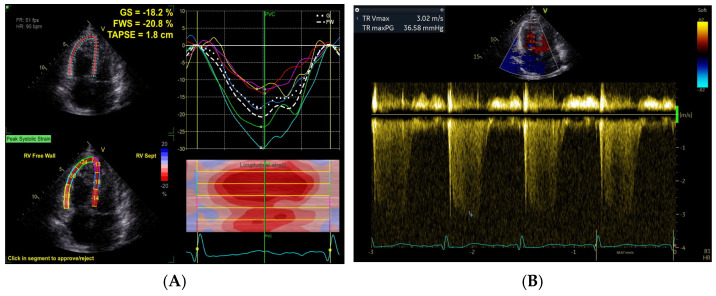
Example of echocardiographic measurement of RVFWLS and PASP: (**A**) Speckle tracking analysis for right ventricular free wall longitudinal strain (RVFWLS, −20.8% in this case), the different colored curves represented strain–time curves of different segments; (**B**) the TR pressure gradient was calculated from the continuous-wave Doppler TR velocity by the simplified Bernoulli equation. PASP was calculated by the formula PASP  =  4  ×  (peak velocity of TR)^2^  +  estimated right atrial pressure (PASP, 52 mmHg in this case). The RVFWLS/PASP ratio was manually calculated as the absolute value of RVFWLS divided by PASP, yielding 0.400%/mmHg in this case.

RV-PA coupling was noninvasively assessed using the RVFWLS/PASP ratio, RVGLS/PASP ratio, and TAPSE/PASP ratio [13]. Two-dimensional grayscale images were acquired from the apical four-chamber view with optimized visualization of the right ventricle. For speckle tracking analysis, three consecutive cardiac cycles were recorded at frame rates of 50–80 frames per second and stored digitally for offline processing. Only images with adequate quality, defined by clear visualization of the endocardial border throughout the cardiac cycle, were included for strain analysis. Right ventricular free wall longitudinal strain was analyzed offline using vendor-specific software (EchoPAC version 204). All strain analyses were performed by experienced analysts who were blinded to patients’ clinical outcomes and baseline risk scores to ensure objective interpretation. After RVFWLS was obtained from the software analysis and PASP was estimated as described above, the RVFWLS/PASP ratio was calculated manually as the absolute value of RVFWLS divided by PASP [14]. A representative example illustrating the key measurements of RVFWLS and PASP is provided in Figure 1. RV global longitudinal strain (RVGLS) was calculated as the average of six RV segments (basal, mid, and apical segments of both the free wall and septum), whereas RV free wall longitudinal strain (RVFWLS) was derived from the average of three RV free wall segments (basal, mid, and apical segments). Both RVFWLS and RVGLS were expressed as absolute values rather than negative values to avoid confusion [15]. TAPSE was measured by M-mode echocardiography with the cursor optimally aligned along the direction of the lateral tricuspid annulus in the apical four-chamber view. To assess the intra- and inter-observer variability, a subset of ultrasound images from randomly selected patients was re-analyzed. For intra-observer variability, the same primary echocardiographer repeated the measurements after an interval of at least two weeks to avoid recall bias. For inter-observer variability, a second independent echocardiographer, who was blinded to the initial measurements, re-analyzed the same images.

### 2.5. Risk Assessment Models

The COMPERA 2.0 four-strata model was applied as described by Boucly et al. [16], utilizing WHO-FC, 6MWD, and NT-proBNP levels for evaluation. Each variable was assigned a score, and the average was calculated by dividing the sum of all scores by the number of variables, then rounded to the next integer to determine the risk stratum. The FPHN noninvasive [17] determined risk stratification by calculating the number of variables achieving low-risk criteria. The REVEAL Lite 2 risk model was implemented according to Benza et al. [18], integrating NT-proBNP, 6MWD, WHO-FC, and estimated glomerular filtration rate for comprehensive assessment.

### 2.6. Variable Selection and Collinearity Assessment

Univariate Cox proportional-hazards regression analyses were used to examine the prognostic relevance of variables that were selected based on clinical significance and guideline recommendations. Variables with *p* < 0.05 were considered for the multivariable Cox proportional hazards model. We employed a backward stepwise elimination procedure to identify independent predictors. However, to ensure clinical validity and robustness, widely recognized prognostic factors (age, sex, and diagnosis) and the main variable of interest (RVFWLS/PASP) were forced into the model regardless of their statistical significance. Our rationale for selecting RVFWLS/PASP is based on the following three points. Firstly, regarding pathophysiological advantage, RVFWLS directly measures the deformation of the right ventricular free wall, which is most sensitive to pressure load. It can more specifically and earlier reflect the impairment of myocardial function compared with RVGLS [19], which includes the interventricular septum, or TAPSE, which only measures annular displacement [20]. Secondly, the existing literature indicates that RVFWLS/PASP performs better than TAPSE/PASP and RVGLS/PASP in risk stratification for pulmonary hypertension [14,21]. Finally, this study aimed to evaluate a novel, more precise echocardiographic indicator of right ventricular–pulmonary artery coupling, and the theoretical foundation of RVFWLS/PASP aligns highly with this objective. RVFWLS/PASP was entered into the stepwise multivariable analysis as a dichotomous variable (<0.246 vs. ≥0.246 %/mmHg). The multicollinearity of the included variables was tested using the variance inflation factor (VIF) method, and VIF < 5 indicated no multicollinearity. Subgroup analyses stratified by etiology were conducted to assess interaction effects, and the corresponding *p*-value for interaction was calculated. The proportional hazards assumption for the final multivariable Cox model was tested using Schoenfeld residuals. A non-significant global test (*p* > 0.05) was considered indicative of no violation of the proportional hazards assumption.

### 2.7. Statistical Analysis

The distribution of continuous variables was assessed using the Shapiro–Wilk test. Based on this assessment, normally distributed variables are presented as mean ± standard deviation and non-normally distributed variables as median (interquartile range). Categorical variables are expressed as numbers (percentages). Group comparisons for normally distributed continuous variables were performed using Welch’s *t*-test or an ANOVA test, and non-normally distributed continuous variables were compared with the Wilcoxon rank-sum test or the Kruskal–Wallis test. Categorical variables were compared between groups using Fisher’s exact test when expected frequencies were below 5; otherwise, the chi-square test was applied. The Spearman correlation coefficient was used to assess relationships between RVFWLS/PASP levels and hemodynamic or laboratory variables.

Restricted cubic spline (RCS) was used to evaluate the relationship between continuous RVFWLS/PASP and primary endpoint. The optimal survival cut-off value of RVFWLS/PASP was determined by maximally selected rank statistics as 0.246%/mmHg, and patients were divided into the higher RVFWLS/PASP group (≥0.246%/mmHg) and the lower RVFWLS/PASP group(<0.246%/mmHg). Survival analysis was conducted using the Kaplan–Meier method with comparisons performed by a log-rank test.

The incremental predictive values of RVFWLS/PASP over the validated risk assessment models including FPHN noninvasive, COMPERA 2.0 four-strata model, and REVEAL Lite 2 risk model were evaluated by calculating net reclassification improvement (NRI). The goodness of fits of the different risk models after incorporating RVFWLS/PASP were assessed using the AIC.

A two-sided *p*-value < 0.05 was considered statistically significant for all analyses. All computations were performed using R (v4.2.2) and IBM SPSS Statistics for Windows (v27.0).

## 3. Results

### 3.1. Baseline Characteristics

A total of 149 patients with PAH were included in this study (Table 1). The cohort comprised 87 patients (58.4%) with IPAH and 62 patients (41.6%) with CTD-PAH. Among all participants, 123 (82.6%) were female. Baseline echocardiography assessment revealed significant RV-PA uncoupling, reduced RV systolic function, and chamber dilation. All patients received standardized medical therapy in accordance with the contemporary clinical guidelines at the time of their diagnosis. Treatment regimens were individualized and optimized during the follow-up period based on risk reassessment and therapeutic response. Analysis of the intra- and inter-observer variability of the RVFW LS/sPAP showed a very good reproducibility.

### 3.2. Relationship Between RVFWLS/PASP and Functional Status in PAH Patients

**Figure 2:**
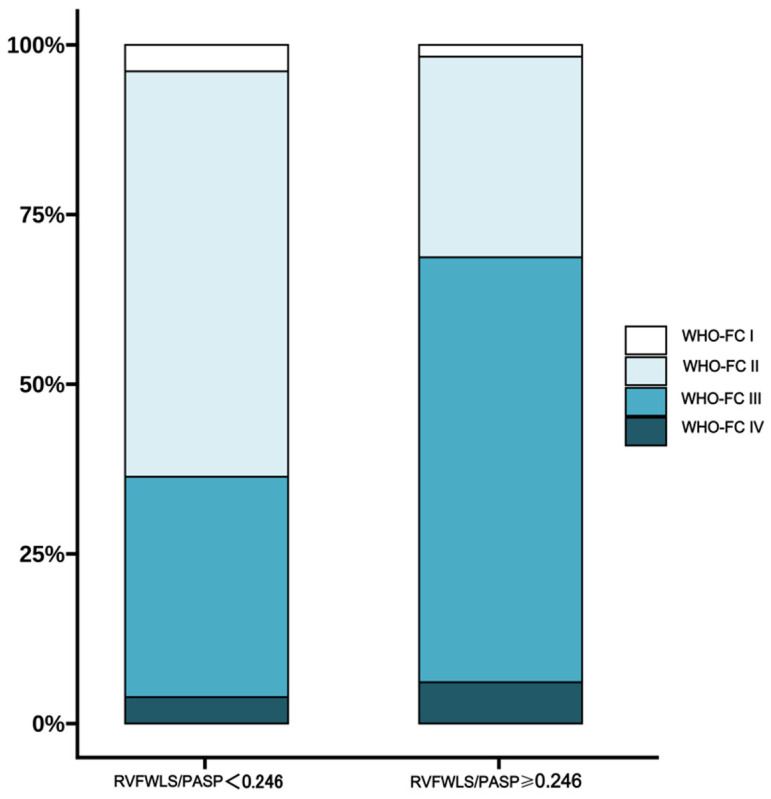
WHO FC in different RVFWLS/PASP groups. RVFWLS/PASP, the RV free wall longitudinal strain to PASP ratio; WHO-FC, World Health Organization functional class.

**Figure 3:**
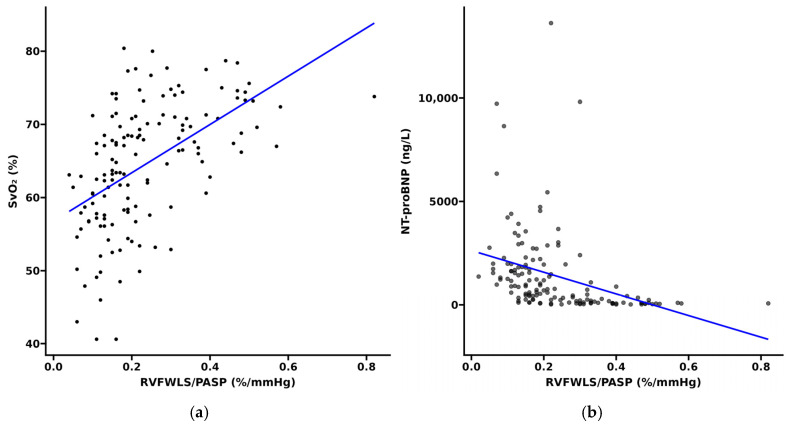
Correlation analysis between RVFWLS/PASP and right heart function: (**a**) Correlation between RVFWLS/PASP and SvO_2_; (**b**) Correlation between RVFWLS/PASP and NT-proBNP. RVFWLS/PASP, the RV free wall longitudinal strain to PASP ratio; NT-proBNP, N-terminal pro-B-type natriuretic peptide; SvO_2_, mixed venous oxygen saturation.

A total of 98 patients were classified into the lower RVFWLS/PASP group (<0.246%/mmHg), while 51 were classified into the higher RVFWLS/PASP group (≥0.246%/mmHg) (Table 1). Compared with the high-ratio group, patients with low RVFWLS/PASP demonstrated significantly impaired exercise capacity and right ventricular function, reflected in reduced 6MWD (375 m vs. 472 m, *p* < 0.001), elevated NT-proBNP (1366 ng/L vs. 97 ng/L, *p* < 0.001), and consistently poorer echocardiographic parameters including RVGLS/PASP (0.14%/mmHg vs. 0.34%/mmHg), TAPSE/PASP (0.20 mm/mmHg vs. 0.37 mm/mmHg), TAPSE (16.2 mm vs. 19.8 mm), and FAC (20% vs. 34%) (all *p* < 0.001). The low-ratio group also exhibited more severe tricuspid regurgitation and right heart dilation, with larger RA area (20 cm^2^ vs. 14 cm^2^), higher LV-EId (1.40 vs. 1.12), and smaller LVEDD (35.7 mm vs. 42.4 mm) despite higher LVEF (78% vs. 72%). Hemodynamically, these patients had elevated pulmonary vascular resistance (PVR) (12.5 WU vs. 6.6 WU) alongside reduced cardiac index (2.51 L/min/m^2^ vs. 3.23 L/min/m^2^). No significant differences were found in diagnostic subtype, sex, age, or BMI between groups. Figure 2 illustrates predominant WHO FC III distribution in the low-ratio group (62.2%) versus WHO FC II predominance in the high-ratio group (66.7%), while correlation analysis (Figure 3) shows RVFWLS/PASP significantly correlated with NT-proBNP (r = −0.64) and mixed venous oxygen saturation (SvO_2_, r = 0.58) (both *p* < 0.001).

### 3.3. Follow-Up Results

The primary composite endpoint was defined as the first occurrence of all-cause mortality, lung transplantation, or rehospitalization for right heart failure. A total of 78 patients (52.3%) reached this endpoint over a median follow-up of 32 months (Table 2). Patients without an event were censored at the last follow-up contact or the study cut-off date (May 2025). Compared with the 71 patients who remained event-free, those who reached the endpoint had more severe baseline impairments, including elevated NT-proBNP levels (median 1276 ng/L vs. 334 ng/L, *p* < 0.001), reduced RV-PA coupling parameters (RVFWLS/PASP: 0.18%/mmHg vs. 0.25%/mmHg; RVGLS/PASP: 0.16%/mmHg vs. 0.23%/mmHg; TAPSE/PASP: 0.22 mm/mmHg vs. 0.27 mm/mmHg; all *p* < 0.05), smaller LVEDD (36.9 ± 6.0 mm vs. 39.2 ± 7.0 mm, *p* = 0.03), elevated mPAP (52 ± 13 mmHg vs. 47 ± 14 mmHg, *p* = 0.03), and increased PVR (10.7 WU vs. 8.4 WU, *p* = 0.01).

### 3.4. Association Between RVFWLS/PASP and Long-Term Outcomes in PAH

**Figure 4:**
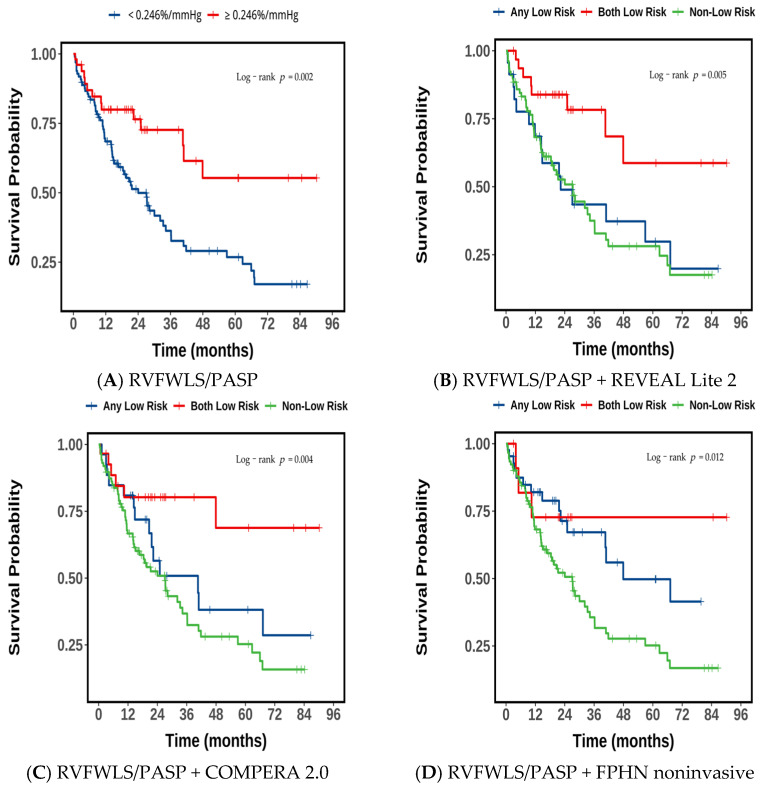
Event-free survival for the composite endpoint according to RVFWLS/PASP and its incremental value over conventional risk models. (**A**) Kaplan–Meier curves for the composite endpoint stratified by RVFWLS/PASP. (**B**–**D**) Incremental prognostic value of RVFWLS/PASP beyond the (**B**) REVEAL Lite 2 model, (**C**) COMPERA 2.0 risk model, and (**D**) FPHN noninvasive model. RVFWLS/PASP was entered into the stepwise multivariable analysis as a dichotomous variable. RVFWLS/PASP, the RV free wall longitudinal strain to PASP ratio; FPHN noninvasive, French Pulmonary Hypertension Network noninvasive; REVEAL = Registry to Evaluate Early and Long-term Pulmonary Arterial Hypertension; COMPERA = Comparative, Prospective Registry of Newly Initiated Therapies for Pulmonary Hypertension. Both Low Risk = low risk by both the conventional risk model and RVFWLS/PASP ratio (<0.246%/mmHg); Any Low Risk = low risk by either the conventional risk model or RVFWLS/PASP ratio (<0.246%/mmHg) alone; Non-Low Risk = Patients not classified as low risk by either single criterion.

As shown in the survival curves (Figure 4A), patients with high RVFWLS/PASP achieved significantly better long-term survival compared with those with low RVFWLS/PASP (log-rank *p* < 0.05). The cumulative survival rates at 1, 3, and 5 years were 80%, 73%, and 55% in the high RVFWLS/PASP group versus 69%, 36%, and 27% in the low RVFWLS/PASP group, respectively.

**Figure 5:**
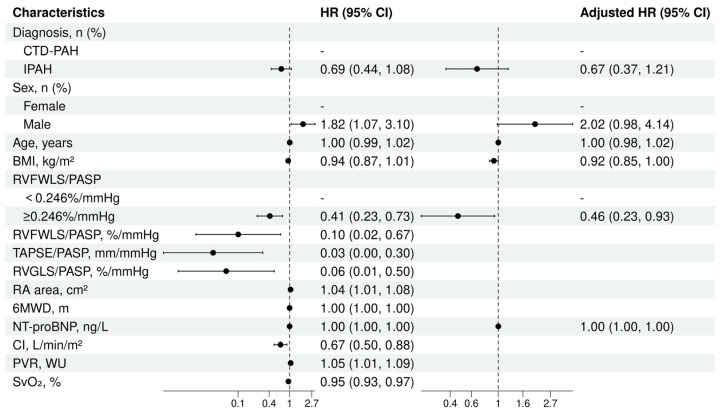
Cox regression analysis for the primary composite endpoint: forest plot of univariate and multivariable hazard ratios. RVFWLS/PASP was entered into the stepwise multivariable analysis as a dichotomous variable. IPAH, idiopathic pulmonary arterial hypertension; CTD-PAH, connective tissue disease-associated pulmonary arterial hypertension; BMI, body mass index; 6MWD, 6 min walking distance; NT-proBNP, N-terminal pro-B-type natriuretic peptide; RVFWLS/PASP, the RV free wall longitudinal strain to PASP ratio; TAPSE/PASP, the tricuspid annular plane systolic excursion to PASP ratio; RVGLS/PASP, the RV global longitudinal strain to PASP ratio; RA area, right atrium area; PVR, pulmonary vascular resistance; SvO_2_, mixed venous oxygen saturation; CI, cardiac index.

Cox regression models were used to evaluate the predictive value of RVFWLS/PASP for the primary composite endpoint. RVFWLS/PASP was analyzed as a dichotomous variable (<0.246 vs. ≥0.246 %/mmHg). Univariate analysis (Figure 5) identified sex, RVFWLS/PASP, RVGLS/PASP, TAPSE/PASP, NT-proBNP, CI, PVR, and SvO_2_ as significant prognostic indicators (all *p* < 0.05). RVFWLS/PASP ≥ 0.246%/mmHg was associated with a reduced mortality rate (HR = 0.41, 95%CI 0.23–0.73, *p* < 0.05). Variables with *p* < 0.05 in the univariate Cox analysis were subsequently included in the multivariate model. Based on clinical relevance and previous literature, age, sex, diagnosis, and RVFWLS/PASP were predefined as mandatory covariates and forced into the model. After excluding collinear variables, we employed a backward stepwise elimination procedure for the remaining candidate variables to determine the final parsimonious model. For the remaining candidate variables, we employed a backward stepwise elimination procedure to determine the final parsimonious model. After adjustment, RVFWLS/PASP ≥ 0.246%/mmHg was independently associated with a reduced mortality (HR = 0.46, 95%CI 0.23–0.93, *p* < 0.05). The proportional hazards assumption was assessed using Schoenfeld residuals. The global test yielded a non-significant result (χ^2^ = 10.92, df = 6, *p* = 0.091), indicating that the proportional hazards assumption was not violated for the final model.

### 3.5. Subgroup Analysis

**Figure 6:**
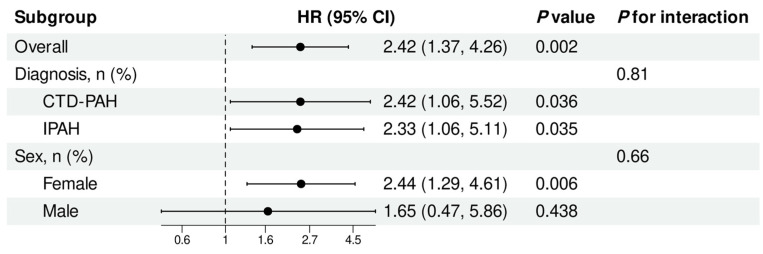
Forest plot of hazard ratios by patient subgroups. IPAH, idiopathic pulmonary arterial hypertension; CTD-PAH, connective tissue disease-associated pulmonary arterial hypertension.

In Figure 6, patients with low RVFWLS/PASP were significantly associated with increased mortality compared with those with high RVFWLS/PASP (HR 2.42, 95% CI 1.37–4.26, *p* = 0.002). This association remained consistent across diagnostic subgroups, with a significant increase in mortality observed in both CTD-PAH (HR 2.42, 95% CI 1.06–5.52, *p* = 0.036) and IPAH (HR 2.33, 95% CI 1.06–5.11, *p* = 0.035) patients. The effect did not significantly differ by primary diagnosis (*p* for interaction = 0.81). Similarly, a significant association was observed in females (HR 2.44, 95% CI 1.29–4.61, *p* = 0.006). The interaction test for sex was not statistically significant (*p* for interaction = 0.66).

### 3.6. Incremental Value of RVFWLS/PASP in Predicting Primary Endpoint

Upon integrating RVFWLS/PASP as a binary variable into three established risk models (FPHN noninvasive, REVEAL Lite 2, and COMPERA 2.0) for Kaplan–Meier survival analysis, the results consistently demonstrated a stratified prognostic pattern. Across all models, patients who simultaneously met the criteria for high RVFWLS/PASP and the respective model’s low-risk category exhibited the most favorable prognosis. In contrast, those with both low RVFWLS/PASP and intermediate-to-high risk according to the respective model had the poorest outcomes. Patients meeting only one of these two criteria consistently showed intermediate survival.

Specifically, in the REVEAL Lite 2 model (Figure 4B), the best combination group (high RVFWLS/PASP + low risk) achieved survival rates of 84%, 78%, and 59% at 1, 3, and 5 years, respectively, while the worst combination group (low RVFWLS/PASP + intermediate–high risk) had rates of 68%, 38%, and 28%. Under the COMPERA 2.0 model (Figure 4C), the best combination group (high RVFWLS/PASP + low risk) demonstrated survival rates of 80%, 80%, and 69%, and the worst combination group (low RVFWLS/PASP + non-low risk) showed rates of 67%, 37%, and 25% for the same time points. In the FPHN noninvasive model (Figure 4D), the best combination group (high RVFWLS/PASP + low risk) had 1-, 3-, and 5-year survival rates all at 73%, whereas the worst combination group (low RVFWLS/PASP + intermediate–high risk) showed rates of 68%, 36%, and 25%, respectively.

Incremental value of RVFWLS/PASP over validated risk models was assessed using the NRI (Table 3). The reclassification ability of risk models was improved as suggested by a significant NRI (all *p* < 0.05). Furthermore, the addition of RVFWLS/PASP to the risk model improved the model’s goodness of fit. Among the models evaluated, the combination of the REVEAL lite 2 score and dichotomous RVFWLS/PASP provided the best fit, as indicated by the lowest AIC value.

## 4. Discussion

Echocardiographic assessment of RV-PA coupling serves as a robust predictor of adverse outcomes in PAH. RVFWLS/PASP derived from two-dimensional speckle tracking echocardiography enables monitoring of RV free wall deformation and represents a more sensitive indicator for evaluating RV function. Our study yielded two principal findings: (1) RVFWLS/PASP emerged as a strong independent predictor of adverse outcomes; and (2) RVFWLS/PASP provided significant incremental predictive value to established prognostic scores.

The rationale for investigating RVFWLS/PASP lies in the biological significance of RVFWLS as a sensitive measure of RV function, providing precise quantification of myocardial fiber deformation. Under pathological conditions, reduced RVFWLS reflects exhaustion of RV contractile reserve, a change that often precedes abnormalities in conventional functional parameters such as TAPSE and FAC [22]. By correcting for afterload using PASP, the RV function is uncoupled from the influence of pulmonary pressure, providing a more precise representation of RV-PA coupling status [23]. The gold-standard measure of RV-PA coupling is the ratio of RV contractility [end-systolic elastance (Ees)] to its load [arterial elastance (Ea)], obtained by conductance catheterization. One study reported a significant correlation between RVFWLS/PASP and Ees/Ea measured by the single-beat method (r = −0.443, *p* = 0.016) [24]. A study by Ancona et al. found that significantly reduced RVFWLS/PASP ratios in patients with severe tricuspid regurgitation were strongly associated with baseline clinical right heart failure (*p* = 0.03) [25]. The optimal survival cut-off value of RVFWLS/PASP was determined by maximally selected rank statistics as 0.246%/mmHg. The cumulative survival rates at 1, 3, and 5 years were 80%, 73%, and 55% in the high RVFWLS/PASP group, versus 69%, 36%, and 27% in the low RVFWLS/PASP group, respectively. This cut-off represents the tipping point of exhausted RV contractile reserve. Below this threshold, patients exhibited significantly reduced exercise capacity and cardiac index, alongside elevated mean pulmonary arterial pressure and pulmonary vascular resistance, indicating inadequate compensation of RV longitudinal contraction for increased pulmonary arterial pressure and manifest mechanical inefficiency [26]. Moreover, future studies incorporating detailed subgroup analyses for each PAH category are warranted [27].

While previous research has established the superiority of the RVFWLS/PASP ratio over conventional coupling parameters for PAH prognosis [28,29], our results further corroborate its clinical relevance. Specifically, RVFWLS/PASP demonstrated a stronger negative correlation with NT-proBNP levels than TAPSE/PASP did (*r* = −0.64, *p* < 0.001 vs. *r* = −0.59, *p* < 0.001). Furthermore, right atrial pressure (RAP) and the pulmonary artery pulsatility index (PAPi) are well-established adverse prognostic factors in patients with PAH [30]. Notably, in the univariate Cox analysis of the present cohort, neither parameter demonstrated independent predictive value (*p* > 0.05). This may be attributed to the relatively limited sample size and the clustered distribution of RAP, which likely reduced their discriminative power. This finding precisely underscores the clinical necessity of exploring novel biomarkers such as RVFWLS/PASP to achieve more precise risk assessment in this patient population.

Although RVFWLS/PASP demonstrates strong predictive potential for PAH [31], it has not yet been incorporated into risk stratification frameworks. Existing risk models such as the FPHN noninvasive model permit use of three noninvasive variables (WHO FC, 6MWD, and BNP/NT-proBNP) to define low-risk status. However, whether adding other noninvasive modalities like echocardiography could further enhance prognostic utility remains unclear. Similarly, COMPERA and REVEAL Lite 2.0 scores primarily rely on clinical and functional assessments while lacking direct evaluation of cardiac structure and function. Recently, Monica et al. reported that RVFWLS/PASP could differentiate between moderate and severe RV dysfunction, thereby refining risk assessment [32]. Notably, in our study, RVFWLS/PASP enabled further stratification of low-risk patients as classified by the FPHN noninvasive, COMPERA, and REVEAL Lite 2.0 scores. Among patients deemed low risk by conventional models, those with high RVFWLS/PASP exhibited superior 1-, 3-, and 5-year survival compared with low RVFWLS/PASP patients. To assess the incremental discriminative value of the RVFWLS/PASP ratio, we systematically compared the NRI between models with and without the RVFWLS/PASP ratio. The results indicate that incorporating the RVFWLS/PASP ratio improves the reclassification ability of the relevant models, highlighting the clinical value of this new variable in risk stratification and supporting its incorporation into the predictive model to improve classification accuracy. Therefore, our findings suggest that integrating echocardiographic RVFWLS/PASP with the FPHN noninvasive, COMPERA, and REVEAL Lite 2.0 scores would yield superior predictive models for adverse outcomes in PAH patients.

## 5. Study Limitations

This study has several limitations. First, its retrospective design and moderate sample size may introduce potential selection bias. Second, a portion of patients without pulmonary artery catheter data were excluded from the study cohort. Nevertheless, this study represents a relatively large-sample investigation of PAH patients with broad inclusion criteria, ensuring the robustness of the findings. In the future, the generalizability and performance of this model require further validation. Furthermore, a limitation of this study is the prolonged follow-up period. During this time, the treatment paradigm for PAH gradually shifted from monotherapy to early combination therapy, constituting an unavoidable potential confounding factor. However, the entire cohort received standardized management in accordance with the prevailing guidelines at the respective times, genuinely reflecting the real-world survival trajectory of PAH. More importantly, the core objective of this study is to evaluate the prognostic value of baseline parameters. Their continued ability to effectively identify potential high-risk populations during subsequent follow-up further highlights the robustness of these indicators in early risk stratification.

## 6. Conclusions

In summary, our study demonstrates that RVFWLS/PASP serves as an independent predictor of long-term prognosis in adults with PAH. Furthermore, RVFWLS/PASP provides incremental predictive value to existing prognostic scoring models, highlighting the importance of assessing right ventricle–pulmonary artery coupling in PAH risk stratification. External validation in independent, larger, multicenter prospective cohorts is warranted in the future.

## Figures and Tables

**Table 1:** Patient demographics and baseline characteristics.

Characteristic	Total *N* = 149	RVFWLS/PASP < 0.246%/mmHg *N* = 98	RVFWLS/PASP ≥ 0.246%/mmHg *N* = 51	*p*-Value
Diagnosis, *n* (%)				0.938
IPAH	87 (58.4%)	57 (58.2%)	30 (58.8%)	
CTD-PAH	62 (41.6%)	41 (41.8%)	21 (41.2%)	
Sex, *n* (%)				0.187
Female	123 (82.6%)	78 (79.6%)	45 (88.2%)	
Male	26 (17.4%)	20 (20.4%)	6 (11.8%)	
Age, years	37 (30, 49)	38 (29, 52)	35 (32, 44)	0.849
BMI, kg/m^2^	21.8 ± 3.3	21.6 ± 3.4	22.1 ± 3.1	0.395
6MWD, m	410 (325, 485)	375 (300, 446)	472 (405, 533)	<0.001
NT-proBNP, ng/L	722 (115, 1812)	1366 (590, 2281)	97 (63, 289)	<0.001
RVFWLS/PASP, %/mmHg	0.19 (0.13, 0.31)	0.15 (0.12, 0.19)	0.37 (0.30, 0.47)	<0.001
TAPSE/PASP, mm/mmHg	0.24 (0.18, 0.33)	0.20 (0.17, 0.24)	0.37 (0.32, 0.48)	<0.001
RVGLS/PASP, %/mmHg	0.17 (0.12, 0.28)	0.14 (0.10, 0.17)	0.34 (0.28, 0.43)	<0.001
TAPSE, mm	17.4 ± 4.0	16.2 ± 3.7	19.8 ± 3.6	<0.001
FAC, %	25 (17, 33)	20 (15, 28)	34 (27, 41)	<0.001
RA area, cm^2^	18 (14, 22)	20 (16, 25)	14 (11, 16)	<0.001
RVMD, mm	35.0 (29.0, 41.0)	38.0 (34.0, 42.0)	27.0 (23.0, 34.0)	<0.001
LV-EId	1.30 (1.13, 1.50)	1.40 (1.23, 1.59)	1.12 (1.00, 1.22)	<0.001
LVEDD, mm	38.0 ± 6.6	35.7 ± 6.0	42.4 ± 5.3	<0.001
LVEF, %	76 ± 9	78 ± 8	72 ± 8	<0.001
E/e′	6.40 (4.92, 8.00)	6.60 (5.10, 8.40)	5.72 (4.65, 7.37)	0.086
mPAP, mmHg	50 ± 14	55 ± 12	39 ± 10	<0.001
PAWP, mmHg	6.0 (4.0, 9.0)	6.0 (4.0, 9.0)	6.0 (4.0, 10.0)	0.523
PVR, WU	10.0 (6.7, 15.3)	12.5 (8.9, 16.8)	6.6 (4.6, 8.7)	<0.001
CI, L/min/m^2^	2.70 (2.23, 3.40)	2.51 (2.07, 3.04)	3.23 (2.70, 3.70)	<0.001
SvO_2_, %	66 (58, 71)	62 (57, 68)	71 (67, 74)	<0.001

Data are presented as mean ± standard deviation, median (interquartile range), or number (percentage). IPAH, idiopathic pulmonary arterial hypertension; CTD-PAH, connective tissue disease-associated pulmonary arterial hypertension; BMI, body mass index; 6MWD, 6 min walking distance; NT-proBNP, N-terminal pro-B-type natriuretic peptide; RVFWLS/PASP, the RV free wall longitudinal strain to PASP ratio; TAPSE/PASP, the tricuspid annular plane systolic excursion to PASP ratio; RVGLS/PASP, the RV global longitudinal strain to PASP ratio; TAPSE, tricuspid annular plane systolic excursion; FAC, RV fractional area change; RA area, right atrium area; RVMD, right ventricular mid-diameter; LV-EId, left ventricular end-diastolic eccentricity index; LVEDD, left ventricular end-diastolic diameter; LVEF, left ventricular ejection fraction; E/e′, ratio of early diastolic mitral inflow velocity to early diastolic mitral annular tissue velocity; mPAP, mean pulmonary arterial pressure; PAWP, pulmonary arterial wedge pressure; PVR, pulmonary vascular resistance; SvO_2_, mixed venous oxygen saturation; CI, cardiac index.

**Table 2:** Baseline characteristics stratified by occurrence of the primary composite endpoint.

Characteristic	Non-Event Group *N* = 71	Event Group *N* = 78	*p*-Value
Diagnosis, *n* (%)			0.065
IPAH	47 (66.2%)	40 (51.3%)	
CTD-PAH	24 (33.8%)	38 (48.7%)	
Sex, *n* (%)			0.058
Male	8 (11.3%)	18 (23.1%)	
Female	63 (88.7%)	60 (76.9%)	
Age, years	38 (30, 48)	35 (30, 52)	0.852
BMI, kg/m^2^	22.1 ± 3.3	21.5 ± 3.3	0.249
6MWD, m	410 (350, 495)	413 (303, 458)	0.302
NT-proBNP, ng/L	334 (89, 1225)	1276 (350, 2281)	<0.001
RVFWLS/PASP, %/mmHg	0.25 (0.14, 0.35)	0.18 (0.13, 0.23)	0.026
TAPSE/PASP, mm/mmHg	0.27 (0.20, 0.37)	0.22 (0.17, 0.28)	<0.001
RVGLS/PASP, %/mmHg	0.23 (0.12, 0.34)	0.16 (0.12, 0.21)	0.027
TAPSE, mm	18.2 ± 4.0	16.7 ± 3.9	0.020
FAC, %	27 (17, 37)	22 (16, 31)	0.066
RA area, cm^2^	17 (13, 21)	18 (15, 24)	0.085
RVMD, mm	34.0 (26.5, 39.5)	36.0 (31.0, 41.0)	0.344
LV-EId	1.24 (1.00, 1.51)	1.31 (1.17, 1.50)	0.394
LVEDD, mm	39.2 ± 7.0	36.9 ± 6.0	0.030
LVEF, %	75 ± 9	77 ± 8	0.331
E/e′	6.40 (5.10, 8.39)	6.20 (4.80, 7.57)	0.471
mPAP, mmHg	47 ± 14	52 ± 13	0.032
PAWP, mmHg	6.0 (4.0, 9.0)	6.0 (3.0, 9.0)	0.231
PVR, WU	8.4 (6.0, 13.8)	10.7 (8.1, 15.5)	0.010
CI, L/min/m^2^	3.04 (2.20, 3.62)	2.64 (2.24, 3.13)	0.045
SvO_2_, %	69 (63, 74)	62 (57, 68)	<0.001

Data are presented as mean ± standard deviation, median (interquartile range), or number (percentage). IPAH, idiopathic pulmonary arterial hypertension; CTD-PAH, connective tissue disease-associated pulmonary arterial hypertension; BMI, body mass index; 6MWD, 6 min walking distance; NT-proBNP, N-terminal pro-B-type natriuretic peptide; RVFWLS/PASP, the RV free wall longitudinal strain to PASP ratio; TAPSE/PASP, the tricuspid annular plane systolic excursion to PASP ratio; RVGLS/PASP, the RV global longitudinal strain to PASP ratio; TAPSE, tricuspid annular plane systolic excursion; FAC, RV fractional area change; RA area, right atrium area; RVMD, right ventricular mid-diameter; LV-EId, left ventricular end-diastolic eccentricity index; LVEDD, left ventricular end-diastolic diameter; LVEF, left ventricular ejection fraction; E/e′, ratio of early diastolic mitral inflow velocity to early diastolic mitral annular tissue velocity; mPAP, mean pulmonary arterial pressure; PAWP, pulmonary arterial wedge pressure; PVR, pulmonary vascular resistance; SvO_2_, mixed venous oxygen saturation; CI, cardiac index.

**Table 3:** Incremental value for prediction and the goodness of fit of the validated risk models after adding RVFWLS/PASP.

Models	AIC	Model Performance Compared with Standard Model	
		NRI (95% CI)	*p*
French Noninvasive	651.8	Ref.	Ref.
+RVFWLS/PASP (≥0.246 mmHg)	642.7	0.253 (0.072–0.433)	0.006
COMPERA 2.0	635.5	Ref.	Ref.
+RVFWLS/PASP (≥0.246 mmHg)	632.4	0.253 (0.209–0.296)	<0.001
REVEAL Lite 2.0	599.2	Ref.	Ref.
+RVFWLS/PASP (≥0.246 mmHg)	597.8	0.253 (0.078–0.427)	0.004

RVFWLS/PASP, the RV free wall longitudinal strain to PASP ratio; AIC, Akaike information criterion; NRI, net reclassification improvement.

## Data Availability

The original contributions presented in this study are included in the article. Further inquiries can be directed to the corresponding author.
